# The COVID-19 pandemic and its implications for cervical cancer treatment and prevention in Zimbabwe: perspectives and recommendations

**DOI:** 10.11604/pamj.2021.39.149.26467

**Published:** 2021-06-25

**Authors:** Grant Murewanhema

**Affiliations:** 1 Unit of Obstetrics and Gynaecology, Faculty of Medicine and Health Sciences, University of Zimbabwe, Harare, Zimbabwe

**Keywords:** COVID-19, pandemic, Zimbabwe, cervical cancer, screening, vaccination, treatment

## Abstract

Cervical cancer is the leading gynaecological malignancy in Zimbabwe, constituting 33% of all female cancers in 2016. Primary prevention through vaccination and secondary prevention through screening are important public health interventions to reduce the cervical cancer burden. Unfortunately, the ongoing COVID-19 pandemic has brought unprecedented challenges to healthcare delivery, posing threats to prevention efforts at a time when the public health sector is extremely fragile. The fragility of the sector has complicated treatment for cervical cancer before and during the COVID-19 pandemic, and is expected to worsen beyond the pandemic. A multi-sectoral intersection between public health experts, clinicians and communities is urgently required to restore preventive and treatment services for cervical cancer and reduce the increased burden, morbidity and mortality stemming indirectly from the pandemic.

## Perspectives

Cervical cancer is the commonest gynaecological malignancy in Zimbabwe [1]. In 2016, according to the Zimbabwe National Cancer Registry, 1308 new cases were reported, and the cancer constituted 33.2% of all female malignancies [1]. Whilst Zimbabwe has a comprehensive cancer registry, reporting may be confounded by failure to access diagnosis and utilisation of services, thus the cervical cancer burden could be much higher than reported [2,3]. The cancer remains a significant public health challenge and a major concern for gynaecologists, oncologists and public health practitioners. Human Papilloma Virus (HPV), the necessary aetiological factor [4] is detectable in over 99% of specimens from patients with histologically confirmed cancer [2,5]. While persistent infection with high-risk HPV genotypes, particularly HPV 16 and 18 causing over 70% of cases, is necessary, failure to access preventive services presents the greatest risk factor for development of invasive cancer [2,3]. Studies have established beyond reasonable doubt the efficacy and safety of HPV vaccines in primary prevention of HPV persistence and premalignant lesions [5-7]. In Zimbabwe, secondary prevention through screening and treatment of precancerous lesions has been the crux of cervical cancer prevention [2]. The country has a high HIV burden, with an estimated 1.3 million people living with HIV/AIDS (PLWHA), about 67% of whom are women [8]. These women are at an additional risk of invasive cervical cancer.

Zimbabwe has been battling the COVID-19 pandemic since reporting the first cases in March 2020 [9]. The cumulative confirmed number of cases had risen to approximately 40,000, with an approximate 1,600 deaths by early June 2021 [10]. Academics have postulated that the actual case burden could be much higher, as poor surveillance and under-testing may be resulting in underreporting. So far, the country has recorded two identifiable waves. The first wave occurred between March 2020 and August 2020, and the second wave occurred December 2020 and January 2021. The second wave was associated with a very rapid increase in case burden and mortality as compared to the first wave. A period of quiescence was observed between February 2021 and May 2021; however, since the beginning of June 2021 the country has started reporting significant increases in the daily reported incident cases, possibly signalling the beginning of a third wave. To contain the spread of SARS-CoV-2, the causative agent for COVID-19, prepare the health system and reduce the adverse socioeconomic impacts associated with increased disease burden and mortality, a myriad of public health interventions were introduced, including lockdowns, travel restrictions and stoppage of all non-essential services, except security and healthcare[9,11]. The restrictions have been introduced in phases dictated by patterns of SARS-CoV-2 transmission [9]; however, access to healthcare in the public sector has remained largely sub-optimal. There has been a slow re-introduction of public health programmes, but the tendency for the outbreaks to occur in waves has meant that these remain subdued or non-existent.

The Zimbabwean government has joined the rest of the world in phased SARS-CoV-2 vaccination, with an objective to inoculate at least 67% of the population to attain herd immunity. By early June 2021, an estimated 400,000 individuals, mainly frontline healthcare and other essential workers had been fully vaccinated, constituting less than 3% of the population [10]. The vaccination landscape faces several challenges, including vaccination hesitancy, limited supply chains and inadequate financial resources, as the country is not part of the COVAX facility. Therefore, it will be a considerable time before vaccination has recognisable population benefits on SARS-CoV-2 transmission and disease outcomes in the country. For now, the World Health Organisation (WHO) recommended infection prevention and control protocols remain the cornerstones for breaking infection chains. Proactive public health practice involves anticipating the short and long-term adverse consequences of the pandemic and its associated restrictive control measures on health outcomes, particularly for programmes that have been considered non-essential and therefore provision stopped or reduced. As Zimbabwe is a high cervical cancer burden country, I discuss the possible indirect impacts of the COVID-19 pandemic on its prevention and treatment in Zimbabwe, the implications for outcomes and possible mitigating public health actions.

**Cervical cancer prevention and treatment in Zimbabwe:** the country has made significant progress towards setting up a national screening programme. Visual inspection methods are available at peripheral health facilities throughout the country, commonly visual inspection with acetic acid cervicography (VIAC). Trained nurses at the centres screen and offer cryotherapy, and refer to higher centres uncertain cases or those suspicious for cervical cancer. At hospitals, conventional Pap smears and colposcopy are offered. Liquid-based cytology, which offers the advantage of reflex HPV deoxyribonucleic acid (DNA) testing, is not yet readily available in the country. Human Papilloma Virus (HPV) DNA testing as primary screening is also not yet available. Patients suspected to have cancer are referred to gynaecologists at higher-level hospitals for diagnosis, evaluation and appropriate treatment, based on stage of disease. Late presentation with very advanced disease is common [2,3]. The country has two gynaecological oncologists, who work at the tertiary Parirenyatwa Group of Hospitals and serve the whole of Zimbabwe. Patients with advanced disease are referred to oncology units, which are constrained with human resources, equipment and medicine shortages. The number of functional radiotherapy machines at any point is limited. Cytotoxic medicines are imported; unfortunately, Zimbabwe is experiencing hyperinflation and foreign currency shortages. Supply chain disruptions due to the COVID-19 pandemic international travel restrictions and suppressed production may have worsened the situation. Vaccination with bivalent HPV vaccines is offered for adolescent girls, supported by the GAVI Alliance. Uptake remains low due to availability and acceptability challenges though significant progress has been made in recent years.

**Impacts of the COVID-19 pandemic on cervical cancer prevention and treatment:** here has been no formal evaluation of the impact of the pandemic on cervical cancer prevention and treatment; however, anecdotal reports point towards reduced utilisation of services. The initial response was total closure of all non-essential services [11]. The lockdown restrictions have been gradually relaxed, but clinical operations remain sub-optimal with facilities operating in emergency mode. Healthcare workers (HCWs) have cited personal protective equipment (PPE) shortages [12]. Emerging evidence from other settings suggests fear and anxiety among HCWs as a significant barrier to service restoration [13]. The COVID-19 pandemic has disrupted global trade and supply chains, leading to shortage of consumables and medicines for screening and treatment [14]. Movement restrictions imposed by lockdowns, coupled with poor roads and networks, may contribute to reduced utilisation of services [12]. Governments and development partners have also temporarily shifted their attention to COVID-19 responses, with possible re-prioritisation and re-allocation of scarce financial resources to pandemic control [11]. An unpublished report has suggested that clients may not seek screening and treatment services during the pandemic for fear of contracting the disease from facilities. Fear of being labelled as COVID-19 cases and community rumours, myths and misconceptions were cited as significant barriers to service utilisation. However, the findings were from qualitative methodologies that have limited external validity and suffered from small sample sizes. Nevertheless, they provided important insights into factors that must be considered at community level to restore normalcy.

**Implications for prevention and treatment:** Zimbabwe, like the rest of the world, is at risk of further waves of COVID-19, and the beginning of June 2021 has seen an increase in the daily incident cases [10]. Due to increasing disease burden, subnormal levels of preventive and treatment services may persist for an unpredictable duration. SARS-CoV-2 vaccination, which is expected to bring some level of normalcy to life and public health delivery, is still grossly off target. In a country whose population remains at considerable risk of HPV acquisition, persistence, emergence of premalignant lesions and development of invasive cancer, several opportunities for prevention are missed. Interruption of HPV vaccination at facilities, and abandonment of targeted vaccination days, mean reduced vaccination coverage. Unfortunately, the COVID-19 pandemic may leave young adolescent girls vulnerable, prone to early marriage and initiation of sexual exposure, leading to early acquisition of HPV infection. Evidence from humanitarian disaster times such as the Ebola outbreak in West Africa points towards adverse sexual and reproductive outcomes for young girls and women as their concerns are neglected [15-17].

The objective of cervical cancer screening is to detect premalignant lesions timeously and offer curative treatment, leading to interception of progression to invasive cancer. A substantial proportion of early-stage, surgically treatable cancers are detected at screening. Missing those results in patients coming in with advanced disease, requiring toxic multimodality treatments, and increased morbidity and mortality. In a country struggling with oncology services due to shortage of specialists and equipment as well as expensive imported cytotoxic drugs, surgery for early stage curable is the best option. The country's public health sector is fragile, and may suffer long-term from the impacts of reduced cervical screening as the burden of invasive cancer may increase substantially. Closure of clinical services implies that patients with established cancer are not getting treatment; unfortunately, cancer growth knows no pandemic [12]. The number of patients to come with metastatic disease and complications including chronic kidney disease, which complicates treatment with concurrent chemoradiation, may increase.

Yearly targeted screening is offered for PLWHA as they present to opportunistic treatment clinics, thus HIV Care and Treatment has been a gateway to prevention for this population. People living with HIV/AIDS may be failing to access services at clinics, and the cost of antiretroviral medicines is expected to increase by 10-25% on the global market [8,18]. Scholarly statistical models point towards an excess of 500,000 HIV-related deaths across Sub-Saharan Africa over the next four years with a 6-month disruption in HIV Care and Treatment [18]. Cervical cancer may contribute to this excess mortality. A health sector that was reported as fragile at the beginning of the pandemic may emerge weaker and fail to cope with increased disease burden [11]. The prevailing health sector challenges in Zimbabwe present significant barriers to cervical cancer treatment and care [3], thus necessitating the need for urgent restoration of preventive screening and vaccination services.

**Recommendations:** restoration of preventive services for women, including cervical cancer screening and HPV vaccination must be prioritised. The WHO has technical guidance on how to restore and maintain essential health services during the pandemic [12,19], which can be contextualised and adapted. Critical to restoration of services is creating an environment that ensures the safety of both HCWs and their clients. Adequate infection prevention and control (IPC) and information, education and communication (IEC) interventions are therefore important domains. Cervical cancer screening messages, alongside other important elements of sexual and reproductive health, must be integrated into IEC material for COVID-19, as the world learns to co-exist with the virus. I suggest some recommendations to restore, maintain or improve cervical cancer prevention and treatment services in Table 1.

**Table 1:** recommendations for restoring cervical cancer screening services

Item	Recommendation
**Infection prevention and control (IPC)**	Provide adequate personal protective equipment to all healthcare workers. Provide adequate and appropriate space that promotes physical distancing for cervical cancer screening. Educate clients and service providers on the need for wearing masks and maintaining appropriate hand hygiene. Continue prioritising vaccination of all healthcare workers to make sure that they are protected from contracting and severe COVID-19. Once possible, ensure widespread population vaccination to reach the target of at least 67% in the shortest possible period of time.
**Information, education and communication (IEC)**	Provide education regarding the need for continued cervical cancer screening through all different forms of media, including social, print and conventional media in languages appropriate for our population. Incorporate cervical cancer screening messages into COVID-19 prevention messages. Widely distribute IEC material to all possible points of access for the population including schools, shops, water distribution points and churches and other religious organisations.
**Accessibility**	Restore all VIAC services in the areas where people live. Encourage outreach programmes for screening and for HPV vaccination for adolescent girls, and restore national vaccination days for vaccine preventable diseases. Provide treatment for precancerous lesions at peripheral facilities and rural and district hospitals through provision of equipment and retraining of nurses, clinical officers and medical officers. Avoid missing opportunities for screening PLWHA during their medicine resupply visits.
**Risk communication and community engagement (RCCE)**	Critical to dispel rumours, myths and misconceptions regarding COVID-19 acquisition in health facilities. Educate the communities that their risk of contracting COVID-19 in the communities may be even higher than that in facilities with adequate IPC measures and therefore they should not be scared of seeking healthcare services.
**Healthcare workers' welfare**	Adequately address workers' remuneration and insurance concerns. Adequately provide them with appropriate PPE.
**Training**	Retrain/refresh healthcare providers on screening and treatment of precancerous lesions and restore their confidence to fearlessly discharge these services.
**Surveillance**	Put in place monitoring and evaluation frameworks to monitor the impact of the pandemic on cervical cancer screening and treatment to adequately inform public health policy and practice.

## Conclusion

Restoration of preventive and treatment services for cervical cancer is urgently needed. Public health experts, clinicians and communities must find an intersection that provides a framework for service restoration to prevent a rise in disease burden and associated morbidity and mortality.
